# Toward Interactions through Information in a Multifractal Paradigm

**DOI:** 10.3390/e22090987

**Published:** 2020-09-04

**Authors:** Maricel Agop, Alina Gavriluț, Claudia Grigoraș-Ichim, Ștefan Toma, Tudor-Cristian Petrescu, Ștefan Andrei Irimiciuc

**Affiliations:** 1 Department of Physics, “Gh. Asachi” Technical University of Iasi, 700050 Iasi, Romania; magop@tuiasi.ro; 2 Romanian Scientists Academy, 54 Splaiul Independentei, 050094 Bucharest, Romania; 3 Department of Mathematics, “Al. I. Cuza” University of Iasi, 700506 Iasi, Romania; 4 Department of Accounting, Audit and Financing, “Stefan cel Mare” University of Suceava, 720229 Suceava, Romania; Claudiag@seap.usv.ro; 5 Department of Material Engineering and Industrial Security, “Gh. Asachi” Technical University of Iasi, 700050 Iasi, Romania; stefan-lucian.toma@academic.tuiasi.ro; 6 Department of Structural Mechanics, “Gh. Asachi” Technical University of Iasi, 700050 Iasi, Romania; tudor.petrescu@tuiasi.ro; 7 National Institute for Laser, Plasma and Radiation Physics, 409 Atomistilor Street, 077125 Bucharest, Romania; stefan.irimiciuc@inflpr.ro

**Keywords:** Shannon information, multifractal theory of motion, Cayley–Klein-type absolute geometries, harmonic mapping, Lobachevsky plane, Poincaré metric

## Abstract

In a multifractal paradigm of motion, Shannon’s information functionality of a minimization principle induces multifractal–type Newtonian behaviors. The analysis of these behaviors through motion geodesics shows the fact that the center of the Newtonian-type multifractal force is different from the center of the multifractal trajectory. The measure of this difference is given by the eccentricity, which depends on the initial conditions. In such a context, the eccentricities’ geometry becomes, through the Cayley–Klein metric principle, the Lobachevsky plane geometry. Then, harmonic mappings between the usual space and the Lobachevsky plane in a Poincaré metric can become operational, a situation in which the Ernst potential of general relativity acquires a classical nature. Moreover, the Newtonian-type multifractal dynamics, perceived and described in a multifractal paradigm of motion, becomes a local manifestation of the gravitational field of general relativity.

## 1. Introduction

In its standard form, the scale relativity theory (SRT) [1,2] employs monofractal dynamics in order to describe complex systems (biostructures, economic systems, etc. [3,4,5]) behaviors. These monofractal dynamics operate through monofractal curves, which are continuous and non-differentiable (being characterized by a single fractal dimension $D_{F}$). Moreover, in the SRT model, a “privileged” fractal dimension exists, namely $D_{F}\to 2$, a situation in which the dynamics of any complex system are described on monofractal manifolds (either through Schrödinger-type geodesics, or through hydrodynamic-type ones). In particular, for dynamics (of complex system entities) on Peano-type curves (i.e., $D_{F}\to 2$) at Compton scale resolutions [1,2], Schrödinger-type geodesics of a monofractal manifold can be identified with the standard Schrödinger equation of quantum mechanics [1,2]. In the same context, hydrodynamic-type geodesics of monofractal manifolds can be identified with the hydrodynamic model of quantum mechanics [1,2]. In such a conjecture, information, in its various forms [6], implies on any type of monofractal manifold [7,8,9,10]:

(i) monofractal tensors of viscous stress, induced by non–differential components of velocity fields;
(ii) uncertainty relationships for constant values of Onicescu’s informational energy [11,12] (values induced through the informational energy maximization principle and assimilated to transitivity manifolds of SL(2R) type groups);
(iii) harmonic mappings from the Euclidian space to hyperbolic plane through the scalar potentials of the velocity fields; and
(iv) “correlation” of the kinetic moments (orbital, spin, or orbital–spin) with “pairs” generation through invariant functions of SL(2R) type groups.

Nature, however, is multifractal at any scale resolution, both from a structural and functional point of view [3,4,5,13,14,15,16]. Thus, it is necessary to extend the SRT, in order to be able to analyze and operate it using multifractal dynamics. This is due to the fact that motion curves belonging to the entities of any complex structure are multifractal curves (i.e., they are continuous and non–differentiable) simultaneously characterized by several fractal dimensions and explicated by the singularity spectrum f(α) (with α being the singularity index [3]). To this end, we present in Section 2, the multifractalization procedure as being part of a “general methodology” of SRT, in the form of the multifractal theory of motion [17,18,19,20,21]. For such a conjecture, in Section 3, we analyze the dynamics of complex systems in the form of Schrödinger- and hydrodynamic-type “regimens” at various scale resolutions. Then, in the same framework, by accepting the principle of Shannon’s information minimization [6,22], we show that for complex system dynamics with radial symmetry, Newtonian-type behaviors dependent on scale resolution can be found (Section 4). The analysis of these behaviors by means of explaining motion geodesics in their conic-type analytical form, using a set of complex variables, specifies the fact that the center of the Newtonian-type multifractal force is different from the center of the multifractal trajectory, the measure of this difference being the eccentricity that depends on the initial conditions (Section 5). In Section 6, the eccentricities’ geometry (i.e., the initial conditions geometry) becomes, through the Cayley–Klein metric principle, the Lobachevsky plane geometry. Then, the harmonic mappings between the usual space and the Lobachevsky plane in a Poincaré metric show that the Ernst potential of general relativity becomes, in essence, of a classical nature. Thus, the Newtonian-type multifractal dynamics, perceived and described through the multifractal theory of motion, becomes a local manifestation of the gravitational field of general relativity, as is required.

## 2. Short Note on the Multifractal Theory of Motion

In what follows, we admit that the motions of the entities of any complex system are described by continuous and non-differentiable curves (multifractal curves). Such a “non–differentiable” procedure to approach these motions has important consequences [1,2,17,18,19,20,21]:

(i) The lengths of multifractal curves tend to infinity when the scale resolution δt tends to zero, according to the Lebesgue theorem [3]. In these conditions, the space becomes a Mandelbrot’s multifractal.
(ii) According to the substitution principle δt≡dt, the complex system dynamics can be corelated to a set of functions during the zoom operation of δt.
(iii) The complex system dynamics are defined by multifractal variables. Under these conditions, two derivatives of any variable field Q(t,dt), which describes the complex system dynamics, can be defined:
  (1) $$\frac{dQ_{+}}{dt}=\underset{\Delta t\to 0}{\lim}\frac{Q\left(t,t+\Delta t\right)-Q\left(t,\Delta t\right)}{\Delta t},\;\frac{dQ_{-}}{dt}=\underset{\Delta t\to 0}{\lim}\frac{Q\left(t,\Delta t\right)-Q\left(t-\Delta t,\Delta t\right)}{\Delta t}.$$
  The sign “+” specifies the forward dynamics. The sign “−” specifies the backward ones.
(iv) The spatial coordinate differential has the form:
  (2) $$d_{\pm }X^{i}\left(t,\;dt\right)=d_{\pm }x^{i}\left(t\right)+d_{\pm }\xi \left(t,\;dt\right)$$
  $d_{\pm }x^{i}\left(t\right)$ does not depend on the scale resolution, while $d_{\pm }\xi \left(t,\;dt\right)$ is scale resolution dependent.
(v) The quantities $d_{\pm }\xi \left(t,\;dt\right)$ satisfy the non–differentiable multifractal equation [3,23,24]
  (3) $$d_{\pm }\xi^{i}\left(t,\;dt\right)=\lambda_{\pm }^{i}{\left(dt\right)}^{\left[\frac{2}{f\left(\alpha \right)}\right]-1}$$
  Details on the physical meanings of the quantities from (3) can be found in [23,24].
(vi) To regain the differential time reflection invariance, we use the operator:
  (4) $$\frac{\hat{d}}{dt}=\frac{1}{2}\left(\frac{d_{+}+d_{-}}{dt}\right)-\frac{i}{2}\left(\frac{d_{+}-d_{-}}{dt}\right).$$
  This is a natural result of Cresson’s theorem [25]. For example, applying the operator (4) to $X^{i}$ yields the complex velocity fields:
  (5) $${\hat{V}}^{i}=\frac{\hat{d}X^{i}}{dt}=V_{D}^{i}-V_{F}^{i}$$
  with
  (6) $$V_{D}^{i}=\frac{1}{2}\frac{d_{+}X^{i}+d_{-}X^{i}}{dt},\;\;V_{F}^{i}=\frac{1}{2}\frac{d_{+}X^{i}-d_{-}X^{i}}{dt},\;\;i=1,2,3.$$
  In this relation, the differential velocity $V_{D}^{i}$ is scale resolution independent, while the non-differentiable one $V_{F}^{i}$ is scale resolution dependent.
(vii) Since the multifractalization describing complex system dynamics implies stochasticization [3,23,24], the whole statistic “arsenal” as of averages, variances, covariances, etc. are operational. Thus, let us select for the average of $d_{\pm }X^{i}$ the subsequent functionality:
  (7) $$\langle d_{\pm }X^{i}\rangle \equiv d_{\pm }x^{i},$$
  with
  (8) $$\langle d_{\pm }\xi^{i}\rangle =0.$$
(viii) To describe the dynamics of complex systems the operator (4) needs to function as a scale covariant derivative [17,18,19]:
  (9) $$\frac{\hat{d}}{dt}=\partial_{t}+{\hat{V}}^{i}\partial_{i}+\frac{1}{4}{\left(dt\right)}^{\left[\frac{2}{f\left(\alpha \right)}\right]-1}D^{lk}\partial_{l}\partial_{k},$$
  where
  (10) $$\partial_{t}=\frac{\partial }{\partial t},\;\;\partial_{i}=\frac{\partial }{\partial X^{i}},\;\;\partial_{l}\partial_{k}=\frac{\partial^{2}}{\partial X^{l}\partial X^{k}}.$$

An example can be given for stochastic processes of Markov-type [3,23,24]

(11) $$\lambda_{+}^{i}\lambda_{+}^{l}=\lambda_{-}^{i}\lambda_{-}^{l}=2\lambda \delta^{il},$$

and

(12) $$f\left(\alpha \right)\equiv D_{F}$$

In (11), monofractal–non-monofractal scale transition can be described through λ coefficient, $\delta^{il}$ being Kronecker’s pseudo-tensor. Then, the scale covariant derivative can be written:

(13) $$\frac{\hat{d}}{dt}=\partial_{t}+{\hat{V}}^{l}\partial_{l}-i\lambda {\left(dt\right)}^{\left[\frac{2}{D_{F}}\right]-1}\partial_{l}\partial^{l}.$$

Moreover, from here, for Peano-type curves, $D_{F}=2$, (13) takes the standard form of the scale covariant derivative from the SRT [1,2]:

(14) $$\frac{\hat{d}}{dt}=\partial_{t}+{\hat{V}}^{l}\partial_{l}-iD\partial_{l}\partial^{l}$$

where λ≡D is the diffusion coefficient attributed to the monofractal–non-monofractal scale transition. Therefore, this model generalizes all the results of Nottale’s theory [1,2]. Moreover, for Compton scale resolution, (14) becomes the “quantum operator” (see [1,2]).

## 3. Dynamics in Complex Systems through Schrödinger- and Hydrodynamic-Type “Regimens” at Various Scale Resolutions

Let us now accept the scale covariance principle (see [1,2]). Then, by applying (9) to (5), the motion equation (the geodesics equation) takes the following form:

(15) $$\frac{\hat{d}{\hat{V}}^{i}}{dt}=\partial_{t}{\hat{V}}^{i}+{\hat{V}}^{l}\partial_{l}{\hat{V}}^{i}+\frac{1}{4}{\left(dt\right)}^{\left[\frac{2}{f\left(\alpha \right)}\right]-1}D^{lk}\partial_{l}\partial_{k}{\hat{V}}^{i}=0.$$

This means that for any complex system dynamics, the multifractal variables (acceleration, $\partial_{t}{\hat{V}}^{i}$, convection, ${\hat{V}}^{l}\partial_{l}{\hat{V}}^{i}$, and dissipation, $D^{lk}\partial_{l}\partial_{k}{\hat{V}}^{i})$ are in equilibrium at any point of the multifractal curve. Particularly, for (11) and (12), the motion equation (the geodesics equation) (15) becomes:

(16) $$\frac{\hat{d}{\hat{V}}^{i}}{dt}=\partial_{t}{\hat{V}}^{i}+{\hat{V}}^{l}\partial_{l}{\hat{V}}^{i}-i\lambda {\left(dt\right)}^{\left[\frac{2}{D_{F}}\right]-1}\partial_{l}\partial^{l}{\hat{V}}^{i}=0.$$

Now, separating the complex system dynamics on scale resolutions, (15) becomes

(17) $$\partial_{t}V_{D}^{i}+V_{D}^{l}\partial_{l}V_{D}^{i}-V_{F}^{l}\partial_{l}V_{F}^{i}+\frac{1}{4}{\left(dt\right)}^{\left[\frac{2}{f\left(\alpha \right)}\right]-1}D^{lk}\partial_{l}\partial_{k}V_{D}^{i}=0\;\partial_{t}V_{F}^{i}+V_{F}^{l}\partial_{l}V_{D}^{i}+V_{D}^{l}\partial_{l}V_{F}^{i}+\frac{1}{4}{\left(dt\right)}^{\left[\frac{2}{f\left(\alpha \right)}\right]-1}D^{lk}\partial_{l}\partial_{k}V_{F}^{i}=0,$$

while (16) takes the form:

(18) $$\partial_{t}V_{D}^{i}+V_{D}^{l}\partial_{l}V_{D}^{i}-\left[V_{F}^{l}+\lambda {\left(dt\right)}^{\left[\frac{2}{f\left(\alpha \right)}\right]-1}\partial^{l}\right]\partial_{l}V_{F}^{i}=0\;\partial_{t}V_{F}^{i}+V_{D}^{l}\partial_{l}V_{F}^{i}+\left[V_{F}^{l}+\lambda {\left(dt\right)}^{\left[\frac{2}{f\left(\alpha \right)}\right]-1}\partial^{l}\right]\partial_{l}V_{D}^{i}=0.$$

For irrotational dynamics (5) become:

(19) $${\hat{V}}^{i}=-\partial^{i}\chi$$

where

(20) $$\chi =-2i\lambda {\left(dt\right)}^{\left[\frac{2}{f\left(\alpha \right)}\right]-1}\ln\Psi$$

In (19) and (20), Ψ is the states function (on the significances of Ψ, see [1,2]), while χ is the scalar potential of (5). By substituting (19) in (16) through specific mathematical procedures [17,18,19], the geodesics equation (16) can be written as a multifractal Schrödinger-type equation:

(21) $$\lambda^{2}{\left(dt\right)}^{\left[\frac{4}{f\left(\alpha \right)}\right]-2}\partial^{l}\partial_{l}\Psi +i\lambda {\left(dt\right)}^{\left[\frac{2}{f\left(\alpha \right)}\right]-1}\partial_{t}\Psi =0.$$

So, through (19), the evolution of any complex system is reflected through multifractal-type Schrödinger “regimens”.

If Ψ is chosen as

(22) $$\Psi =\sqrt{\rho }e^{is},$$

with $\sqrt{\rho }$ the amplitude and s is the phase, (19) takes the explicit form:

(23) $${\hat{V}}^{i}=2\lambda {\left(dt\right)}^{\left[\frac{2}{f\left(\alpha \right)}\right]-1}\partial^{i}s-i\lambda {\left(dt\right)}^{\left[\frac{2}{f\left(\alpha \right)}\right]-1}\partial^{i}\ln\rho .$$

From here, two real velocities result:

(24) $$V_{D}^{i}=2\lambda {\left(dt\right)}^{\left[\frac{2}{f\left(\alpha \right)}\right]-1}\partial^{i}s$$

(25) $$V_{F}^{i}=\lambda {\left(dt\right)}^{\left[\frac{2}{f\left(\alpha \right)}\right]-1}\partial^{i}\ln\rho .$$

By (24) and (25), according to the methodology from [17,18,19], the geodesics Equation (21) becomes the multifractal hydrodynamic-type equations:

(26) $$\partial_{t}V_{D}^{i}+V_{D}^{l}\partial_{l}V_{D}^{i}=-\partial^{i}Q$$

(27) $$\partial_{t}\rho +\partial_{l}\left(\rho V_{D}^{l}\right)=0$$

with Q as the specific multifractal potential:

(28) $$Q=-2\lambda^{2}{\left(dt\right)}^{\left[\frac{4}{f\left(\alpha \right)}\right]-2}\frac{\partial^{l}\partial_{l}\sqrt{\rho }}{\sqrt{\rho }}=-V_{F}^{i}V_{F}^{i}-\frac{1}{2}\lambda {\left(dt\right)}^{\left[\frac{2}{f\left(\alpha \right)}\right]-1}\partial_{l}V_{F}^{l}.$$

Equation (26) is the specific multifractal-type momentum conservation law, while Equation (27) is the multifractal-type states density conservation law. Through (28), the specific multifractal force:

(29) $$F^{i}=-\partial^{i}Q=-2\lambda^{2}{\left(dt\right)}^{\left[\frac{4}{f\left(\alpha \right)}\right]-2}\partial^{i}\frac{\partial^{l}\partial_{l}\sqrt{\rho }}{\sqrt{\rho }}$$

becomes a measure of motion curve multifractality.

Thus, through (26)–(28), the complex system dynamics can described through hydrodynamic “regimens” of a multifractal type:

The following consequences result:

(i) Any complex system’s entities are in a continuous interaction with a multifractal medium through (29);
(ii) Any complex system can be identified with a multifractal fluid. In such conditions, its dynamics are described by the multifractal hydrodynamic model (Equations (26)–(28));
(iii) The velocity field $V_{F}^{i}$ does not represent the contemporary dynamics. It contributes to the transfer of the specific multifractal-type momentum and to the multifractal-type energy focus. This can be easily observed from the absence of $V_{F}^{i}$ from (27) and also from its role in the multifractal variational principles (for details see [3]); and
(iv) If a multifractal-type tensor is chosen:
  (30) $${\hat{\tau }}^{il}=2\lambda^{2}{\left(dt\right)}^{\left[\frac{4}{f\left(\alpha \right)}\right]-2}\rho \partial^{i}\partial^{l}\ln\rho$$
  the equation defining the multifractal-type “forces” that derive from a multifractal-type “potential” Q can be written in the form of a multifractal-type equilibrium equation. This equation can be written in a tensorial form:
  (31) $$\rho \partial^{i}Q=\partial_{l}{\hat{\tau }}^{il}.$$
  where
  (32) $${\hat{\tau }}^{il}=\eta \left(\partial_{l}V_{F}^{i}+\partial_{i}V_{F}^{l}\right)$$
  and
  (33) $$\eta =\lambda {\left(dt\right)}^{\left[\frac{2}{f\left(\alpha \right)}\right]-1}\rho .$$

Thus, (31) is a linear constitutive equation of a multifractal type for a multifractal-type “viscous fluid”. Moreover the η coefficient can be assimilated to a multifractal-type dynamic viscosity of a multifractal-type fluid.

## 4. Information and Multifractal Interactions

Let us consider a positive probability density $\rho \left(x\right)=\psi \left(x\right)\bar{\psi }\left(x\right)$ and a finite set of constraints:

(34) ∫ρ(x)dx=1

(35) $$\int f_{k}\left(x\right)\rho \left(x\right)dx={\bar{f}}_{k},\;\;k=1,\;\ldots ,n.$$

Now, we can find a probability density ρ(x), which minimizes Shannon’s information [22]:

(36) $$H\left(\rho ,m\right)=\int \rho \left(x\right)\ln\frac{\rho \left(x\right)}{m\left(x\right)}dx$$

bound to conditions (34) and (35). Note that (36) can be invariant to any transformation group through quantity m(x). Usually, the quantity m(x) is identified with the invariant function of the transformation group selected for analysis. Thus, if the group selected for analysis is SL(2R), then the integral invariant function is unitary [26]. Then, from a stochastic point of view, it can be stated that the variables of this group are uniformly distributed, a situation in which the principle that minimizes the transformation (36) is identified with that of the maximum Onicescu’s informational energy E(ρ) [11]:

(37) $$E\left(\rho \right)=\int \rho^{2}\left(x\right)dx.$$

In this conjecture, a standard method for finding the minimum of (36) is to use the Lagrange multipliers method [27], corresponding to certain given constraints, in order to obtain the functional expression:

(38) $$I\left(\rho ,m\right)=\int \rho \left(x\right)\ln\frac{\rho \left(x\right)}{m\left(x\right)}dx+\beta \int \rho \left(x\right)dx+\overset{m}{\underset{1}{\sum }}\lambda_{m}\int f_{k}\left(x\right)\rho \left(x\right)dx.$$

By equaling with zero the variation of this functional expression, with respect to ρ(x), the following equation will be obtained:

(39) $$\ln\frac{\rho \left(x\right)}{m\left(x\right)}+1+\beta +\overset{m}{\underset{1}{\sum }}\lambda_{k}f_{k}\left(x\right)=0$$

By solving it with respect to ρ(x), we can find:

(40) $$\rho \left(x\right)=m\left(x\right)exp\left[-\lambda_{0}-\overset{m}{\underset{1}{\sum }}\lambda_{k}f_{k}\left(x\right)\right]$$

where the notation $\lambda_{0}=\beta +1$ has been made.

The minimum variation of information can now be expressed with respect to the multipliers $\lambda_{k}$ and the values ${\bar{f}}_{k}$, by multiplying (39) with ρ(x) and integrating. We obtain:

(41) $$H_{min}\left(\rho ,m\right)=-\lambda_{0}-\overset{m}{\underset{1}{\sum }}\lambda_{k}{\bar{f}}_{k}$$

Now it is necessary to choose $\lambda_{0}$ and $\lambda_{k}$, in such a way that the constraints (34) and (35) may be identified.

Under constraint (34), for $\lambda_{0}$, the following value results:

(42) $$\lambda_{0}=ln\int m\left(x\right)exp\left[-\overset{m}{\underset{1}{\sum }}\lambda_{k}f_{k}\left(x\right)\right]dx$$

which implies the dependence:

(43) $$\lambda_{0}=ln\;F\left(\lambda_{1},\ldots ,\;\lambda_{m}\right).$$

In order to obtain under a finite form the function $F\left(\lambda_{1},\ldots ,\;\lambda_{m}\right)$, in the cases when the integration from (42) can be carried out, the values $\lambda_{1},\ldots ,\;\lambda_{m}$ can be found through the equation:

(44) $$-\frac{\partial }{\partial \lambda_{k}}\lambda_{0}={\bar{f}}_{k}$$

which results from (42) and (35).

Unfortunately, it is usually impossible to solve equations (42) and (44) in a finite form, in order to explicitly provide $\lambda_{k}$. There are, however, cases in which these operations can be carried out, and these are the ones in which the a priori density is exponential. Indeed, if m(x) is a multivariant exponential:

(45) $$m\left(x\right)=\frac{1}{a_{1}\ldots \;a_{n}}exp\left[-\sum \frac{x_{k}}{a_{k}}\right]$$

where $x=(x_{1},\ldots ,\;x_{m})$ with $x_{k\;}$ real and positive, and if the constraints are:

(46) $$\int x_{k}\rho \left(x\right)dx={\bar{x}}_{k},\;\;k=1,\;\ldots ,n$$

then the system (44) can be immediately solved and leads to the a posteriori density:

(47) $$\rho \left(x\right)=\frac{1}{{\bar{x}}_{1}\ldots \;{\bar{x}}_{n}}exp\left[-\sum \frac{x_{k}}{{\bar{x}}_{k}}\right].$$

Thus, the density remains exponential, but with parameters $a_{k}$ being substituted with the averages extracted from the constraints (46).

This is a “convenient” example in which, depending on the nature of the constraints, the a priori and a posteriori densities are the same. However, the exponential can be obtained as a posteriori distribution when it is known that the a priori distribution is uniform. Let us look at the case of a single variable, uniformly distributed on any interval of the positive half–line-segment.

Imposing the restrictions:

(48) ∫ρ(x)dx=1

(49) $$\int x\rho \left(x\right)dx=\bar{x}$$

for minimizing the function:

(50) H(ρ,1)=∫ρ(x)lnρ(x)dx

the exponential is obtained:

(51) $$\rho \left(x\right)=\frac{1}{\bar{x}}exp\left(-\frac{x}{\bar{x}}\right).$$

This can be, for example, the case of density ρ(x) for which x is the radial distance r (i.e., x≡r) and $\bar{x}$ is the average radial distance $r_{0}$ (i.e., $\bar{x}\equiv r_{0})$. Then, through (51), it results in:

(52) $$\rho \left(r\right)=\frac{1}{r_{0}}exp\left(-\frac{r}{r_{0}}\right).$$

Now, in the case of the radial symmetry r, we can obtain, by substituting this result in the expression of the specific multifractal potential (28), the following:

(53) $$Q\left(r\right)=-2\lambda^{2}{\left(dt\right)}^{\left[\frac{4}{f\left(\alpha \right)}\right]-2}\frac{1}{\sqrt{\rho }}\left(\frac{d^{2}\sqrt{\rho }}{dr^{2}}+\frac{2}{r}\frac{d\sqrt{\rho }}{dr}\right)=-2\lambda^{2}{\left(dt\right)}^{\left[\frac{4}{f\left(\alpha \right)}\right]-2}\left(\frac{1}{4r_{0}^{2}}-\frac{1}{r_{0}r}\right)$$

and also through (29) the multifractal specific force:

(54) $$F\left(r\right)=-\frac{2\lambda^{2}{\left(dt\right)}^{\left[\frac{4}{f\left(\alpha \right)}\right]-2}}{r_{0}r^{2}}.$$

## 5. Multifractal Dynamics of a Newtonian-Type

In what follows, we present a few of the characteristics of the motion induced by the multifractal force field (54) by using the method described in [21]. By means of this, the results from the abovementioned reference are expanded upon for any scale resolution (be it atomic, infra-galactic, extra-galactic, etc.).

In the case when the inertial effects of a multifractal type, $\partial_{t}V_{D}^{i}$, are dominant with respect to the convective effects of multifractal-type, $V_{D}^{l}\partial_{l}V_{D}^{i}$, meaning that the condition $\left|\partial_{t}V_{D}^{i}\right|\gg \left|V_{D}^{l}\partial_{l}V_{D}^{i}\right|$ is satisfied, the specific multifractal-type momentum conservation law (18), with the constraint (54), is given, in a vectorial notation, by the equation:

(55) $$\ddot{r}+\frac{k}{r^{3}}\;r\;=0\;$$

with

(56) $$k=\frac{2\lambda^{2}{\left(dt\right)}^{\left[\frac{4}{f\left(\alpha \right)}\right]-2}}{r_{0}}$$

Here, k is a physical constant that depends both on the scale resolution given by $\lambda^{2}{\left(dt\right)}^{\left[\frac{4}{f\left(\alpha \right)}\right]-2}$ as well as on the shielding length $r_{0}$, r is the position vector, and $\ddot{r}$ is the acceleration vector.

A vectorial multiplication of (55) with r has, as a consequence, the essential characteristic of force (54): it generates only planar motions (the planar motion is kept):

(57) $$\frac{d}{dt}\left(r\times \dot{r}\right)=r\times \ddot{r}=0$$

Due to the existence of a plane of motion, it is possible to simplify the geometry of the problem by limiting it to the said plane, in which the coordinates of an “entity” in motion are ξ and η.

Then, (55) becomes equivalent to the system:

(58) $$\ddot{\xi }+\frac{k}{r^{2}}\cos\Phi =0,\;\;\ddot{\eta }+\frac{k}{r^{2}}\sin\Phi =0$$

Here, r and Φ are the polar coordinates in the plane of motion relative to the attraction center. The magnitude of the velocity, which is involved in the variation of the area “swept” by r, is of the form:

(59) $$\dot{a}=\xi \dot{\eta }-\eta \dot{\xi }=r^{2}\Phi$$

The integration of system (58) through (59) leads to the analytical form of the trajectory. First, the complex variable is defined:

(60) $$X_{3}=\xi +i\eta =re^{i\Phi }$$

Then, (58) becomes:

(61) $$\ddot{X_{3}}+\frac{k}{r^{2}}e^{i\Phi }=0$$

Now, (59) can be used in order to eliminate $r^{2}$, which implies:

(62) $$\ddot{X_{3}}+\frac{k}{\dot{a}}e^{i\Phi }\dot{\Phi }=0∴\dot{X_{3}}=i\left(\frac{k}{\dot{a}}e^{i\Phi }+w\right)$$

where:

(63) $$w=w_{1}+iw_{2},\;i=\sqrt{-1}$$

is a complex constant that is obviously determined through initial conditions. The analytic equation of motion is obtained from (59) and the second equation (62), which in polar coordinates of the plane of motion, leads to the expression:

(64) $$\frac{\dot{a}}{r}=\frac{k}{\dot{a}}+w_{1}\cos\Phi +w_{2}\sin\Phi$$

or, in (ξ,η) coordinates:

(65) $$\left(\frac{k^{2}}{{\dot{a}}^{2}}-w_{1}^{2}\right)\xi^{2}-2w_{1}w_{2}\xi \eta +\left(\frac{k^{2}}{{\dot{a}}^{2}}-w_{2}^{2}\right)\eta^{2}+2\dot{a}\left(w_{1}\xi +w_{2}\eta \right)={\dot{a}}^{2}$$

This result is a conic. The center of the conic does not coincide with the center of the force, rather it has the coordinates given through the initial conditions:

(66) $$\xi_{0}=-\frac{\dot{a}w_{1}}{\Delta },\;\;\eta_{0}=-\frac{\dot{a}w_{2}}{\Delta },\;\;\Delta ={\left(\frac{k}{\dot{a}}\right)}^{2}-w_{1}^{2}-w_{2}^{2}\;\;$$

For Δ=0, the geometric center of the trajectory is located at infinity and the trajectory becomes a parabola.

Assuming, however, that the center of the trajectory is located at a finite distance and relating the geometric description of the trajectory to said center through the translation:

(67) $$X_{1}=\xi -\xi_{0},\;\;X_{2}=\eta -\eta_{0}$$

the trajectory equation becomes:

(68) $$\left(\frac{k^{2}}{{\dot{a}}^{2}}-w_{1}^{2}\right)X_{1}^{2}-2w_{1}w_{2}X_{1}X_{2}+\left(\frac{k^{2}}{{\dot{a}}^{2}}-w_{2}^{2}\right)X_{2}^{2}=\frac{k^{2}}{\Delta }$$

This is still a conic, with the difference that the relationship is related to its center and orientation (i.e., the direction of its center with respect to the center of the multifractal force) is given by the vector $\left(w_{1},w_{2}\right)$. In such a situation, the supplementary condition Δ>0, which shows that the vector $\left(w_{1},w_{2}\right)$ is limited in modulus, specifies the fact that the trajectory is not an ellipse anymore, but rather a hyperbola or a parabola.

It is obvious that in (65), the issue shifts toward a conic for which the line:

(69) $$w_{1}\xi +w_{2}\eta -\dot{a}=0$$

is the polar of the multifractal force center. This lends direct kinematic significance to the coordinates of the conic center, which are determined through the initial motion conditions. As such, the following temporal characterization of the trajectory can be given: an entity (i.e., a particle) launched with any initial velocity in the space of a center that exerts a multifractal attraction force inversely proportional to the square of the distance, eventually describes a conic in the plane of motion. The initial velocity of the entity determines the relative position of the center of motion with respect to the center of the multifractal force. If the magnitude of this velocity is under a certain limit, then the trajectory is an ellipse. Otherwise, this trajectory is either a parabola or a hyperbola. Moreover, as a result from above, the center of the multifractal force is different from the center of the multifractal trajectory, the measure of this difference being the eccentricity, which depends on the initial conditions. A set of such eccentricities can be found, all of them depending on the initial conditions and reflecting all the “possibilities” of realization of Newtonian-type multifractal dynamics.

## 6. Geometries Involved in Multifractal Dynamics of a Newtonian-Type

The set of conics can be structured as a Cayleyan space, in which the squared forms from the conics’ equations in Cartesian coordinates are represented through points with coordinates given by their coefficients. The absolute of this space is given in the context of form (65) of the trajectory equation, by the conic:

(70) $$\frac{k^{2}}{{\dot{a}}^{2}}-w_{1}^{2}-w_{2}^{2}=0$$

This conic represents a circle in the plane of velocity motions induced by the multifractal force field (54). The points inside the circle represent in absolute geometry ellipses, and relate to initial velocities that are upper-bounded in magnitude by a certain value dictated by the multifractal force field (54). The points on the absolute represent parabolas, and the points outside the absolute represent hyperbolas. Let it be noted that the nature of the trajectory in the multifractal forces field (54) is dictated by the initial velocity of the entity and that, for the current trajectory to be an ellipse, the initial velocity must be upper-bounded in modulus. The geometry of the initial velocities plane is a hyperbolic geometry and it is a natural geometry of the velocity space in special relativity [28].

Thus, the families of trajectories in the multifractal force field (54) can be systematically characterized through a non-Euclidean geometry of the relative positions of the motion centers, represented by these trajectories with respect to the unique center of the multifractal forces field (54). First of all, the parabolic trajectories are all represented through points on the circle (70), which can be written in the form:

(71) $$\mu^{2}+\nu^{2}=1,\;\;\mu =e\cos\Omega ,\;\;\nu =e\sin\Omega$$

where e represents the eccentricity of the trajectory, defined by relations:

(72) $$a^{2}=\frac{k^{2}}{\Delta^{2}},\;\;b^{2}=\frac{{\dot{a}}^{2}}{\Delta },\;\;e^{2}=\frac{a^{2}-b^{2}}{a^{2}}{\left(\frac{\dot{a}}{k}\right)}^{2}\left(w_{1}^{2}+w_{2}^{2}\right)\;\;$$

with a and b are the half-axis of the orbit. Thus, the initial condition can actually be expressed only in terms of “contemporary” magnitudes, allowing the discarding of previous considerations:

(73) $$w_{1}=\frac{k}{\dot{a}}e\cos\Omega ,\;\;w_{2}=\frac{k}{\dot{a}}e\sin\Omega$$

The absolute metric of the interior of the circle (71) has the expression:

(74) $$ds^{2}=\frac{\left(1-\nu^{2}\right){\left(d\mu \right)}^{2}+2\mu \nu d\mu d\nu +\left(1-\mu^{2}\right){\left(d\nu \right)}^{2}}{1-\mu^{2}-\nu^{2}}$$

and can be distilled to a form given by Poincaré for the metric of the hyperbolic plane, or by one given by Lobachevsky:

(75) $$ds^{2}=-4\frac{dhd\bar{h}}{{\left(h-\bar{h}\right)}^{2}}=-\frac{du^{2}+dv^{2}}{v^{2}}$$

through the following coordinates transformations:

(76) $$\mu =\frac{h\bar{h}-1}{h\bar{h}+1},\;\;\nu =\frac{h+\bar{h}}{h\bar{h}+1}$$

(77) $$h=u+iv=\frac{\nu +i\sqrt{1-\mu^{2}-\nu^{2}}}{1-\mu },\;\;\bar{h}=u-iv$$

The metric (75) is a differential invariant of a continuous group with three parameters (a group of SL(2R)-type) [17,18] of the complex plane, whose infinitesimal generators are the Killing vectors of the metric.

In short, the projection of the “momentum” forms generated by the metric on these Killing vectors are differential forms, which represent constant rates along the metric geodesics. The three Killing vectors have the expressions [17,18]:

(78) $${\hat{B}}_{1}=\frac{\partial }{\partial u},\;\;{\hat{B}}_{2}=u\frac{\partial }{\partial u}+v\frac{\partial }{\partial v},\;\;{\hat{B}}_{3}=\left(u^{2}-v^{2}\right)\frac{\partial }{\partial u}+2uv\frac{\partial }{\partial v}$$

Now, the two components of the “momentum” vector can be obtained by considering the metric (75) as a Lagrangian of the geodesic motion. This provides, for the “momentum”, the differential forms:

(79) $$p_{u}=\frac{du}{v^{2}},\;\;p_{v}=\frac{dv}{v^{2}}$$

By projecting this vector on the Killing vectors from (78), the differential forms result:

(80) $$\omega_{1}=\frac{du}{v^{2}},\;\;\omega_{2}=2\frac{udu+vdv}{v^{2}},\;\;\omega_{3}=\frac{\left(u^{2}-v^{2}\right)du+2uvdv}{v^{2}}$$

Then, it is easy to check that the metric (75) can be reproduced by the squared expression:

(81) $$\frac{1}{4}\left(\omega_{2}^{2}-4\omega_{1}\omega_{3}\right)$$

and that the differential forms from (80) are proportional with the elementary arch of the geodesics of metric (75), geodesics given by the parametric equations:

(82) $$u\left(s\right)=u_{0}+v_{0}\tanh\left(v_{0}s\right),\;\;v\left(s\right)=\frac{v_{0}}{\cosh\left(v_{0}s\right)}$$

with $u_{0}$ and $v_{0}$ constant.

Now, in terms of parameters (e,Ω), the metric (74) becomes:

(83) $$ds^{2}={\left(\frac{de}{1-e^{2}}\right)}^{2}+\frac{e^{2}}{\left(1-e^{2}\right)}{\left(d\Omega \right)}^{2}$$

Since for elliptic trajectories “e” varies in the interval between −1 and +1, the substitution:

(84) e=tanhχ

in (83) implies:

(85) $$ds^{2}={\left(d\chi \right)}^{2}+\sinh^{2}\chi {\left(d\Omega \right)}^{2}$$

The parameter h from (77) has a direct relationship with the Ernst potential of general relativity [28,29], by means of harmonic mapping.

In order to showcase this relation, the parameter h is rewritten in terms of (e,Ω). The expression is:

(86) $$h=i\frac{\cosh\theta +\sinh\theta e^{-i\Omega }}{\cosh\theta -\sinh\theta e^{-i\Omega }},\;\;\chi =2\theta$$

It happens that (81) represents a harmonic map for the Euclidian space into the hyperbolic plane, if χ (and θ) represents a solution of the Laplace equation in free space:

(87) Δχ=0

For details on the problem of harmonic correspondences between the usual space and the Lobachevsky plane, see [28,30].

Under this conditions, the energy functional stationary values are corelated to the solutions of the Euler–Lagrange equations for a Lagrangian such as:

(88) $$L=-4\frac{\gamma^{il}\partial_{i}h\partial_{l}\bar{h}}{{\left(h-\bar{h}\right)}^{2}}$$

with $\gamma^{il}$ the metric of the usual space. Then, through

(89) $$\delta ∭\frac{\gamma^{il}\partial_{i}h\partial_{l}\bar{h}}{{\left(h-\bar{h}\right)}^{2}}\sqrt{det\left(\gamma \right)}d^{3}x$$

it results:

(90) $$\left(h-\bar{h}\right)\partial^{l}\partial_{l}h=2\partial_{l}h\partial^{l}h$$

and its complex conjugate. Then, it can be seen that h from (86) verifies (90), when χ is a solution of the Laplace equation and Ω is arbitrary (it does not depend on the position space). In the case h≡iε, the variational principle (89) in the form (Ernst principle [28,29,30]):

(91) $$\delta ∭\frac{\gamma^{il}\partial_{i}\varepsilon \partial_{l}\bar{\varepsilon }}{{\left(\varepsilon +\bar{\varepsilon }\right)}^{2}}\sqrt{det\left(\gamma \right)}d^{3}x=0$$

refers exclusively to the complex potential of Ernst [28,29] (i.e., to the gravitational field in vacuum).

**Figure 1:**
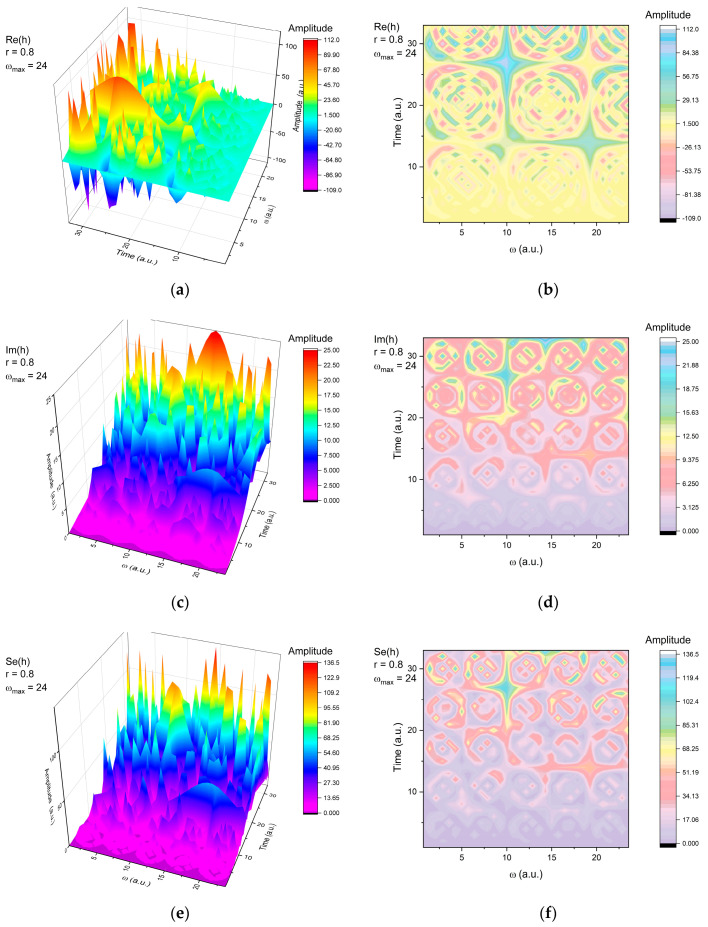
3D representation and contour plot for the real (Re(*h*)-(**a**,**b**)) and imaginary (Im(*h*)-(**c**,**d**)) part of *h* as well as the full representation of the signal (Se(*h*)-(**e**,**f**)).

In what follows, let us explain the harmonic mapping modes, based both on scale resolution and temporal ordering. In Figure 1, we show the 3D representation (left side) and contour plot (right side) for the real (Re(*h*)) and imaginary (Im(*h*)) part of *h* as well as the full representation of the signal (Se(*h*)), where $r=\tanh\left(\frac{\chi }{2}\right)$, Ω=ωt and ω is the pulsation of motion and t is the time. It is possible to see a multi-structuring and modulation of the signal in all three representations. The real part of *h* (Re(*h*)) presents three structures in both time and ω coordinate while the Im(*h*) presents multiple similar structures that are seen to blend together for a short amount of time. When representing the complete function (Se(*h*)), we can observe that the structure follows the patterns imposed by the implicit contribution on the oscillatory dynamics through Im(*h*), while the nature of the oscillations is given by the explicit contribution through Re(*h*).

**Figure 2:**
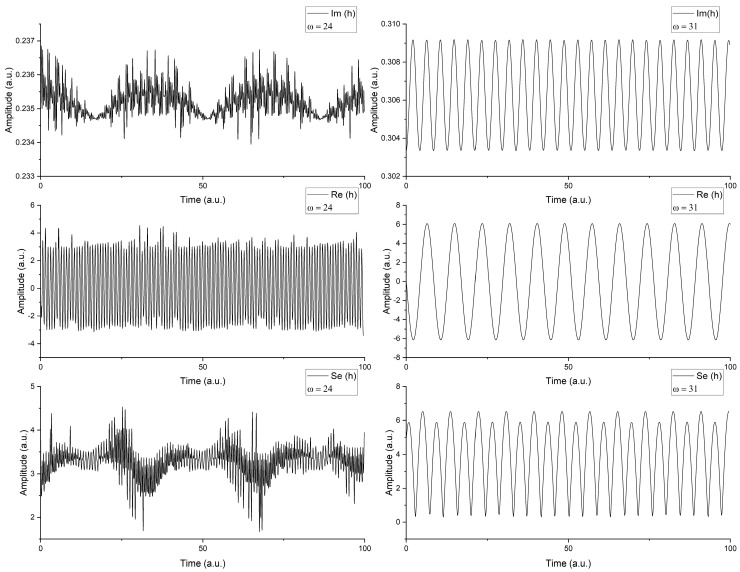
Time series of the Re(*h*), Im(*h*), and Se(*h*) for two different scale resolutions (ω = 24, left side and ω = 31, right side).

Thus, we can find three different representations of the function that reflect the explicit (Re(*h*)), implicit (Im(*h*)), and the measurable (Se(*h*)) contributions. When looking for the impact of the external factors, through the control parameter, *r*, it is possible to notice that various dynamics can be induced at different scale resolutions (ω_max_). In Figure 2, we have represented the time-series of the three aforementioned functions for a fixed control parameter, *r* = 0.8, which will depict a dynamic unperturbed by external factors for two different scale resolutions (ω = 24, left side and ω = 31, right side). It can be observed that the period doubling is actually a superposition of the real and imaginary part of *h*. The real and imaginary parts depict a classical oscillatory behavior with different frequencies. Changes in the scale resolution would automatically be reflected by the shape and structure of the time series (right-hand side of Figure 2). One can observe a complicated oscillatory structure in the imaginary plane, where a modulated structure overlapping an intermittent oscillatory dynamic can be seen, while in the real plane, a high frequency oscillatory structure can be noticed. These fascinating results come from the representation in the measurable plane, which is here seen as quasi-chaotic and mostly following the structure imposed by the Im(*h*). This means that for some systems in which the explicit form is not chaotic, non-linear behaviors can transpire from the implicit information where chaos can be “hidden”.

**Figure 3:**
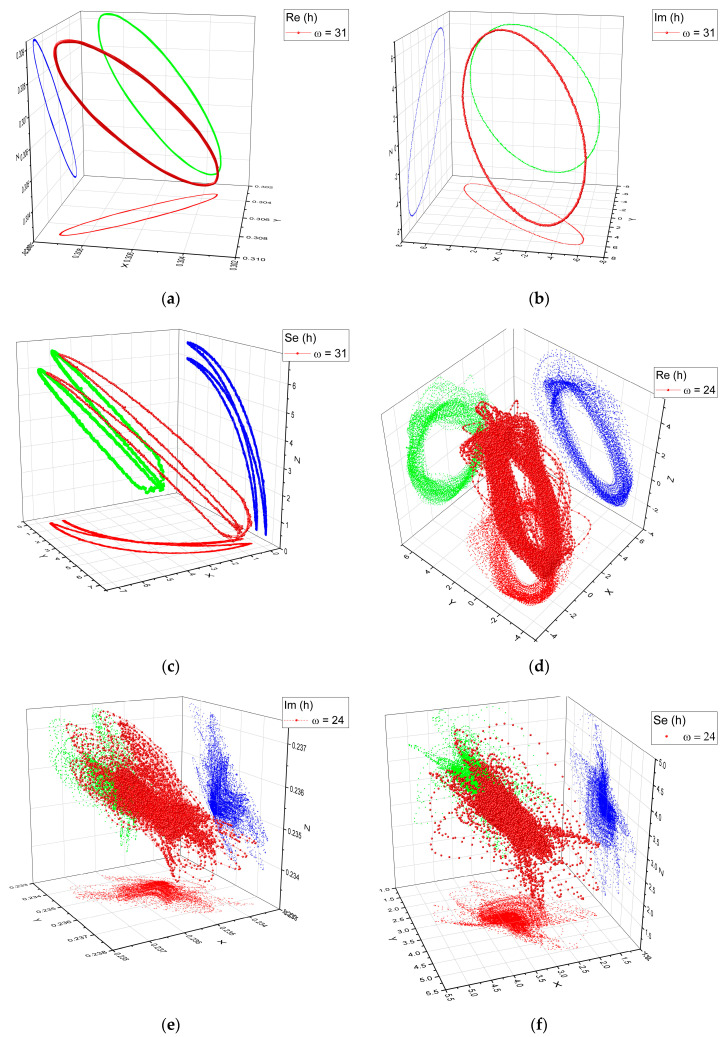
Reconstruction of the system attractors in the phase space for the Re(*h*), Im(*h*) and Se(*h*) at two resolution scales (ω = 24: (**d**–**f**) and ω = 31: (**a**–**c**)).

In order to quantify or even showcase the implicit chaos, as suggested above, the attractors for all of the time series represented in Figure 3 have been reconstructed. For ω = 31, it was possible to observe single frequency oscillations, with the difference being in the “flatness” of the attractor in response to the changes in the frequencies between the real and imaginary part. The Se(*h*) attractor showcased the double period structure. For ω = 31, a quasi-oscillatory behavior for the Re(*h*) was noticed, while the Im(*h*) presented a chaotic structure with multiple trajectories seen in the projections in the XZ or XY planes. This attractor seems to describe wide trajectories converging to a “focal area”. The Se(*h*) attractor depicted similar trajectories as in the case of Im(*h*), the main difference being that the higher density of trajectories was at the base of the attractor, becoming scarcer toward the edge of the attractor.

## 7. Conclusions

The main conclusions of this paper are as follows:

(i) The specific monofractal dynamics of Nottale’s scale relativity theory were expanded to multifractal dynamics in the form of the multifractal theory of motion. Consequently, a short presentation of the non-differentiability in the multifractal theory was given.
(ii) A quick overview of Shannon’s information was made (a) to define this information and its invariance regarding any transformation group; (b) from a stochastic point of view, if the variables of the transformation group are uniformly distributed, then the principle that minimizes the information is identified with that of the maximum Onicescu’s informational energy; (c) the Lagrange multipliers method is used in the minimization of information; (d) the explicitation of the information in the case of a complex system exhibiting dynamic radial symmetry is given; and (e) the correlation between Shannon’s information “subjected” to the “qualities” described in (a)–(d) and the multifractal theory of motion in its hydrodynamic form implies Newtonian-type behaviors dependent on scale resolution (i.e., interactions of a multifractal Newtonian-type).
(iii) The analysis of these behaviors in the framework of the multifractal theory of motion in its hydrodynamic form involves, through a set of complex variables, motion geodesics (i.e., trajectories) with their analytic expression given by means of conics. In such a context, the result is that the center of the Newtonian-type multifractal force is different from the center of the multifractal trajectory, the measure of this difference being the eccentricity that depends on the initial conditions.
(iv) The eccentricities’ geometry (the initial conditions geometry), becomes, through the Cayley–Klein metric principle, the Lobachevsky plane geometry. Then, harmonic mappings between the usual space and the Lobachevsky plane in a Poincaré metric means that the Ernst potential of general relativity becomes, in essence, of a classical nature. So, this represents a harmonic application from the usual space to the matter–field space, without making use of the concept of material structure, so necessary for the general relativity theory in Einstein’s view. Thus, a “physics” of the initial conditions “specified” by Newtonian-type multifractal motions becomes functional, to which it corresponds to a Lobachevsky geometry through the Cayley–Klein metric principle. Through this, the algebraic structure associated with these “physics” is isomorphic with the algebraic structure “dictated” by the Ernst principle upon the gravitational field in vacuum. As such, the gravitational field revealed in two separate instances—multifractal theory of motion (see (89)) and general relativity (see (91))—presents a joint group invariant of SL(2R)-type, which can be determined by means of Stoka’s procedure (for details see [17,21]). In such a context, the Newtonian-type multifractal dynamics perceived and described through the multifractal theory of motion become a local manifestation of the gravitational field of general relativity, as is required.
(v) Several types of harmonic mappings, given by both scale resolution and temporal ordering, were graphically explained.
(vi) The present theoretical model becomes applicable to any case of the existence of a real conic, being easy to demonstrate that the Cayleyan metric attached to this conic is a metric generated by the transformation group of SL(2R)-type, which leaves it invariant.
